# Diagnostic performance of Contrast-enhanced CT in Pyrrolizidine Alkaloids-induced Hepatic Sinusoidal Obstructive Syndrome

**DOI:** 10.1038/srep37998

**Published:** 2016-11-29

**Authors:** Xuefeng Kan, Jin Ye, Xinxin Rong, Zhiwen Lu, Xin Li, Yong Wang, Ling Yang, Keshu Xu, Yuhu Song, Xiaohua Hou

**Affiliations:** 1 Department of Radiology, Union Hospital, Tongji Medical College, Huazhong University of Science and Technology, Wuhan, 430022, P. R. China; 2 Division of Gastroenterology, Union Hospital, Tongji Medical College, Huazhong University of Science and Technology, Wuhan, 430022, P. R. China

## Abstract

Hepatic sinusoidal obstruction syndrome (HSOS) can be caused by pyrrolizidine alkaloids(PAs)-containing herbals. Since PAs exposure is obscure and clinical presentation of HSOS is unspecific, it is challenge to establish the diagnosis of PAs-induced HSOS. Gynura segetum is one of the most wide-use herbals containing PAs. The aim of our study is to describe the features of contrast-enhanced computed tomography (CT) in gynura segetum-induced HSOS, and then determine diagnostic performance of radiological signs. We retrospectively analyzed medical records and CT images of HSOS patients (71 cases) and the controls (222 cases) enrolled from January 1, 2008, to Oct 31, 2015. The common findings of contrast CT in PAs-induced HSOS included: ascites (100%), hepatomegaly (78.87%), gallbladder wall thickening (86.96%), pleural effusion (70.42%), hepatic vein narrowing (87.32%), patchy liver enhancement (92.96%), and heterogeneous hypoattenuation (100%); of these signs, patchy enhancement and heterogeneous hypoattenuation were valuable features. Then, the result of diagnostic performance demonstrated that contrast CT possessed better performance in diagnosing PAs-induced HSOS compared with various parameters of Seattle criteria. In conclusion, the patients with PAs-induced HSOS display distinct radiologic features at CT-scan, which reveals that contrast-enhanced CT provides an effective noninvasive method for diagnosing PAs-induced HSOS.

Hepatic sinusoidal obstruction syndrome (HSOS) (also named veno-occlusive disease or VOD) is defined as a non-thrombotic obstruction of the sinusoids without thrombosis or other underlying disorders of the hepatic veins (HVs)^1^,^2^. Sometimes, HSOS occurs after the ingestion of pyrrolizidine alkaloids (PAs) which are contained in about 3% of the world’s flowering plants^3^. One of the most wide-use herbals containing PAs is gynura segetum^4^,^5^,^6^,^7^. Clinical presentations of HSOS include jaundice, right upper-quadrant pain and tender hepatomegaly, ascites, and unexplained weight gain. A detailed history of PAs exposure and clinical presentations define PAs-induced HSOS in patients. Since clinical presentations of HSOS mimic other liver disorders, the PAs exposure is essential for the diagnosis of HSOS. However, it is difficult to detect PAs in dietary supplements or herbals because PAs are found in quiet a lot of herbals and the component of herbals is complex. Recently, an ultra-performance liquid chromatography-mass spectrometry assay was used to determine pyrrole-protein adducts in peripheral blood^5^,^6^,^8^, which provided a useful approach to establish the diagnosis of PAs-induced HSOS. However, the assay does not apply to clinical practice due to high expense and unavailability in most hospital. Therefore, it is challenge to establish the diagnosis of HSOS caused by PAs-containing herbal because PAs exposure can be obscure and clinical presentations of HSOS are unspecific. Given this, it is important to generalize an non-invasive mean to differentiate HSOS from other liver diseases.

Utility of radiological imaging in the diagnosis of liver diseases has been confirmed in clinical practice. Recently, several studies have described the CT/MRI features of PAs-induced HSOS based on a small number of the patients^4^,^9^,^10^; but diagnostic value of radiological imaging should be investigated due to small sample size and the lack of the controls in previous studies^4^,^9^,^10^. In view of this, the purpose of our study is to retrospectively describe the features of contrast-enhanced CT in PAs-induced HSOS, and then to determine diagnostic performance of contrast CT.

## Results

### The characteristics of the patients

**Figure 1:**
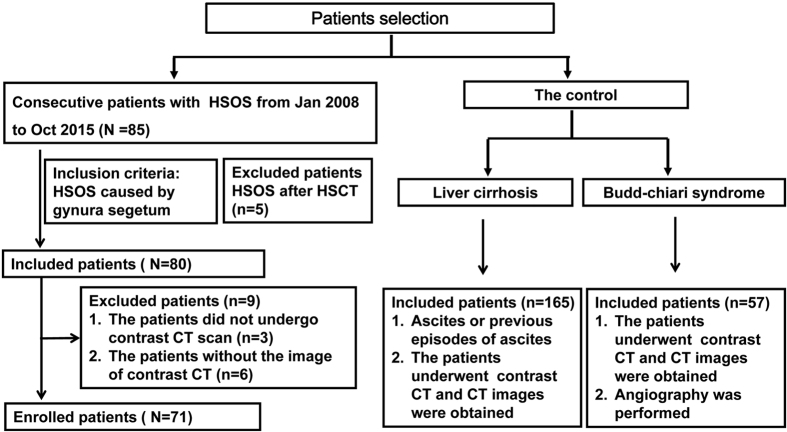
Flowchart of the patients selection, inclusion, and exclusion.

In this study, the study population consisted of 293 patients with a diagnosis of PAs-induced HSOS (N = 71), BCS (N = 57), and liver cirrhosis (N = 165) (Fig. 1). Demographic and biochemical characteristics of the included patients were summarized in Table 1. Firstly, clinical features of these patients were evaluated. As expected, abdominal distension resulted mainly from ascites and right upper quadrant pain were common clinical manifestations of PAs-induced HSOS. Whereas a small proportion of PAs-induced HSOS patients had weight gain and edema. Secondly, the results of blood routine examination showed that the values of erythrocyte, leukocyte and platelet were in normal range in most of the PAs-induced HSOS patients. Thirdly, biochemical parameters of the patients were also determined. The results of liver function tests showed that biomarkers of liver damage (ALT, AST) and cholestasis (ALP, γGT) exceeded the upper limit of normal range in most of the HSOS patients; moreover, albumin and PT, biochemical parameters associated with synthetic ability of liver, were also abnormal in most of PAs-induced HSOS patients (Table 1). Simultaneously, the result of blood routine examination showed erythrocyte, leukocyte and platelet were in normal range in most of BCS patients; conversely, anemia, leukopenia and thrombocytopenia were observed in more than half of cirrhotic patients due to hypersplenism secondary to portal hypertension. The BCS patients had better liver function than HSOS, which was revealed by liver function test (Table 1).

### Radiological findings of contrast-enhanced CT in HSOS patients

**Figure 2:**
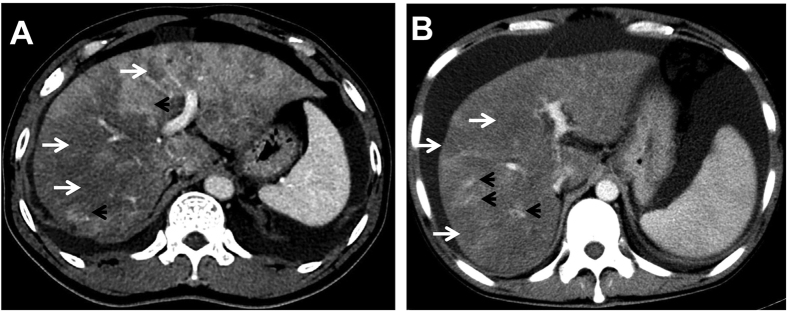
Contrast-enhanced CT scans showed that heterogeneous hypoattenuation and patchy liver enhancement were two most important radiological signs of PAs-induced HSOS. Heterogeneous hypoattenuation represented heterogeneous hypoattenuated, or low-density areas appear darker (arrow); patchy liver enhancement was inhomogeneous enhancement of liver parenchyma (arrowhead).

**Figure 3:**
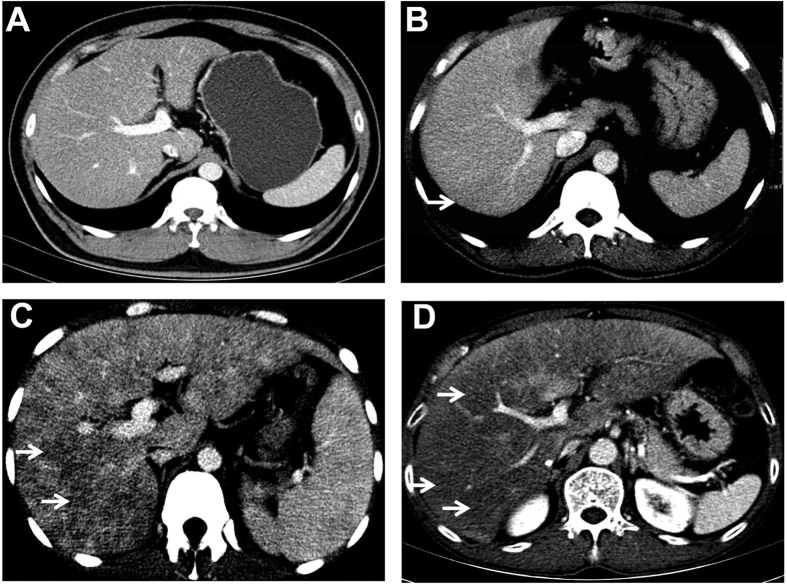
The severity of heterogeneous hypoattenuation on CT images: (**A**) equivocal, (**B**) mild, (**C**) moderate, and (**D**) severe heterogeneous hypoattenuation in the liver. Arrow: heterogeneous hypoattenuation. Because heterogeneous hypoattenuation is obvious in portal phase, the data were obtained from portal phase CT scan.

**Figure 4:**
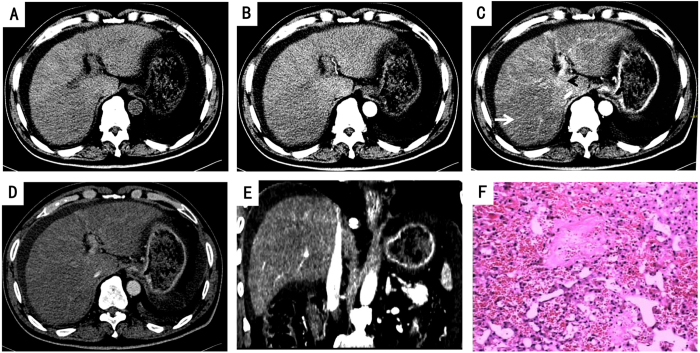
67-year-old man diagnosed with gynura segetum-induced HSOS received contrast-enhanced CT scan and liver biopsy. (**A**–**D**) Image of plain and contrast-enhanced CT scan; (**A**) plain CT scan; (**B**) arterial phase; (**C**) portal phase; (**D**) equilibrium phases. The CT image in portal phase showed patchy liver enhancement (arrowhead,) and heterogeneous hypoattenuation (arrow) in PAs-induced HSOS. (**E**) Multiple planar reconstruction CT angiogram shows a normal patent inferior vena cava despite it was slightly compressed by swelling liver. (**F**) Pathologic examination showed that demonstrates sinusoidal congestion and dilation, and extensive extravasation of erythrocytes into pericentral parenchyma.

Tables 2 illustrated radiographic signs seen on contrast-enhanced CT in HSOS patients or the controls. Firstly, we analyze the features of pre-contrast CT in the patients with HSOS or the controls. Ascites (100%) and the global enlargement of the liver (78.87%) were common radiologic findings of HSOS in plain CT, which was consistent with clinical features of HSOS. In addition, most of the HSOS patients presented with gallbladder wall thickening (86.96%) and pleural effusion (70.42%). As shown in Table 2, these radiologic features were more common in HSOS patients than the controls (liver cirrhosis or BCS). Secondly, we further analyze the imaging findings of contrast CT. On contrast-enhanced CT, patchy liver enhancement (92.96%) and heterogeneous hypoattenuation (100%) are two most common radiologic findings in PAs-induced HSOS (Figs 2, 3 and 4). Patchy liver enhancement denotes inhomogeneous enhancement of liver parenchyma (Fig. 2); heterogeneous hypoattenuation represents heterogeneous low-density areas which appear darker on CT scan (Figs 2 and 3). A small proportion of BCS patients presented patchy liver enhancement (28.07%) and heterogeneous hypoattenuation (19.30%). None of these findings was seen in the group of liver cirrhosis. All these indicated that heterogeneous hypoattenuation in portal phase was the most important feature of PAs-induced HSOS on contrast-enhanced CT. In view of this, the severity scores based on the extent of heterogeneous hypoattenuation were evaluated. Interobserver agreement between the two radiologists for scoring severity was excellent (k = 0.932, 95% confidence interval: 0.891–0.973), and the consensus severity scores were used in the data analysis. The severity of heterogeneous hypoattenuation in PAs-induced HSOS patients was indicated by grade B in 12 patients (16.90%), grade C in 26 patients (36.62%), grade D in 33 patients (46.48%). Simultaneously, 46 (80.70%), 4 (7.02%), 6 (10.53%) and 1 (1.75%) cases were scored as grade A, grade B, grade C and grade D, respectively in BCS group (Table 2). It revealed that heterogeneous hypoattenuation in HSOS group was obvious both in the incidence and in the severity compared with BCS patients. To further differentiate PAs-induced HSOS from BCS, homogeneous in equilibrium phase was analyzed. Homogeneous in equilibrium phase was present in 6 (8.45%) of the 71 patients with PAs-induced HSOS and in 8 (50%) of the 16 subjects of BCS. Finally, we also investigated other radiologic findings of contrast CT. The image findings associated with portal hypertension such as collateral circulation and splenomegaly were less common in the HSOS patients compared with the control (BCS or liver cirrhosis) (Table 2). Meanwhile, another interesting sign of HSOS was hepatic vein narrowing, which presented in 87.32% of the PAs-induced HSOS patients.

### Diagnostic performance of contrast CT or various parameters of Seattle criteria

In order to further evaluate diagnostic performance of radiologic findings, the sensitivity, specificity, positive predictive value (PPV), negative predictive value (NPV) and accuracy were calculated (Table 3). Firstly, diagnostic efficacy of various parameters of Seattle criteria was determined. Ascites achieved an overall sensitivity of 100%, a specificity of 31.98%, a PPV of 31.98%, a NPV of 100%, and an accuracy of 48.46%; the respective values for jaundice were 50.7%, 61.93%, 30.25%, 79.41%, and 59.17%; the respective values for hepatomegaly were 78.87%, 83.33%, 60.22%, 92.5%, and 82.25% for the diagnosis of HSOS. Then, we determined diagnostic efficacy of valuable radiologic signs: patchy liver enhancement and heterogeneous hypoattenuation. The radiologic finding of patchy liver enhancement yielded a sensitivity of 92.96%, a specificity of 92.79%, a PPV of 80.49%, a NPV of 97.63%, and an accuracy of 91.83%; the respective values for heterogeneous hypoattenuation were 100%, 95.05%, 86.59%, 100%, and 96.25%. These indicated that diagnostic value of radiologic findings was superior to that of various parameters of Seattle criteria. All these indicated that contrast-enhanced CT was effective in diagnosing PAs-induced HSOS.

## Discussion

HSOS can be caused by ingestion of herbs containing PAs^4^,^5^,^6^,^7^. The Seattle and Baltimore criterion used for the diagnosis of VOD/HSOS are primarily based on clinical findings that include: painful hepatomegaly, hyperbilirubinemia, and fluid retention^11^,^12^. Since PAs-induced HSOS is a potentially life threatening diseases, early detection of HSOS should be a priority. Seattle criteria was more sensitive in detecting of PAs-induced HSOS compared with Baltimore criteria^13^. In view of this, several studies defined PAs-induced HSOS using Seattle criteria^4^,^5^,^6^,^8^. Thus, our study used Seattle criteria in defining PAs-induced HSOS. Then, we determined diagnostic performance of Seattle criteria and the result demonstrated that various parameters of Seattle criteria had limited diagnostic efficacy. The diagnosis of HSOS can be established by liver biopsy, with histological findings including sinusoidal dilatation and congestion, hepatocyte necrosis. However, liver biopsy was not performed in most of gynura segetum-induced HSOS due to thrombocytopenia, clotting abnormalities and extensive ascites. The patchy nature of HSOS incurs sampling variability that confines diagnostic yield of biopsy^14^. In addition, clinical value of transvenous liver biopsy is limited due to its high rate of false negatives, high expense and limited availability. More importantly, sinusoidal congestion and hepatocellular necrosis are also observed in BCS and congestive hepatopathy^15^. In conclusion, all these confined the application of liver biopsy in diagnosing PAs-induced HSOS. In this study, we described radiologic findings of gynura segetum-induced HSOS on contrast CT based on a relatively large cases. Patchy liver enhancement and heterogeneous hypoattenuation in contrast-enhanced portal phase CT scans were valuable signs. Further study showed that contrast-enhanced CT possessed better diagnostic efficacy than that of various parameters of Seattle criteria. It indicated that contrast-enhanced CT provided an effective noninvasive method for the diagnosis of PAs-related HSOS.

Previous studies has demonstrated MRI techniques, such as SPIO-enhanced T2-weighted GRE images and gadoxetic acid-enhanced magnetic resonance imaging identified HSOS in the patients of colorectal cancers who had received oxaliplatin-based chemotherapy^14^,^16^,^17^. Given the difference in the cause of HSOS, radiological features of oxaliplatin-induced HSOS might be hardly extrapolated to PAs-induced HSOS. In consider of this, the image features of MRI should be described; unfortunately, most of enrolled patient with PAs-induced HSOS in our study did not receive MRI scan; therefore, we did not provide the data on MRI in PAs-indcued HSOS.

Patchy enhancement and heterogeneous hypoattenuation were also observed in a small proportion of BCS; fortunately, there is significant difference in the proportion and the severity between BCS and PAs-induced HSOS. Careful assessment of HVs and IVC provides assistance in differentiating BCS from PAs-induced HSOS; in addition, splenomegaly, collateral hepatic venous circulation, reversed or turbulent flow, and regenerative nodules can be indicative of BCS. For PAs-related HSOS, detailed history of PAs exposure provides a direct evidence. Previous studies demonstrated that patchy enhancement was an important sign of BCS^18^,^19^,^20^. However, our study showed that a small proportion (28.07%) of BCS patients presented inhomogeneous with a mottled appearance in liver. Some important factors gave rise to the difference between our results and previous reports^18^,^19^,^20^. Firstly, some of BCS patients in previous studies were diagnosed by clinical manifestations, laboratory tests, and imaging modalities (ultrasonography, CT, MRI); whereas, the BCS patients in our study received percutaneous angiography which was considered as the “gold standard”^20^. Secondly, ethnic difference might cause the difference in radiologic findings. Additionally, we further analyzed the causes which resulted in a small proportion of BCS patients with a mottled appearance. Heterogeneous patchy enhancement was observed in acute phase^19^,^20^; however, most of the BCS patients in our study were chronic cases, which were indicated by splenomegaly, portosystemic collateral circulation, and onset time. Moreover, we also found that fewer of chronic BCS patients with IVC lesions had heterogeneous patchy enhancement compared with BCS patients with the lesions in HVs. Collateral circulation, such as spleno-renal shunts and intrahepatic portosystemic shunts, alleviated the congestion of the liver, and then heterogeneous patchy enhancement was reduced accordingly.

Did HSOS induced by different causes present similar signs on enhanced CT? In the patients who had undergone oxaliplatin-based chemotherapy, the most common feature of enhanced CT was heterogeneous hypoattenuation of the hepatic parenchyma on CT or MRI scan^14^,^16^,^17^,^21^. In HSOS patients (12 cases) after HSCT, periportal edema, ascites, and a narrow right hepatic vein were common findings of CT; while, heterogeneous hypoattenuation and patchy enhancement were not observed^22^. In HSOS patient (1 case) associated with tacrolimus following liver transplantation or the patient (1 case) who used a recreational drug during anal intercourse, patchy enhancement, heterogeneous hypoattenuation were observed^9^,^23^. All these indicated that the radiologic signs of contrast-enhanced CT were various due to the different causes in HSOS. On the other hand, the severity of HSOS might lead to the difference in radiologic findings of contrast-enhanced CT.

Obviously, our study has several limitations, including its retrospective nature. Firstly, pathological evidences were not available in most of the patients (58/71; 81.69%) due to ascites, thrombocytopenia, and coagulation disorders. Therefore, we could not correlate the histological change in the liver with the CT images. However, histological assessment based on one block of tissue and heterogeneous pathological distribution in HSOS limited the study on the correlation between the histological change and image findings. Secondly, the images collected from three CT scanners may result in the bias in reviewing radiologic signs. Finally, cardiac cirrhosis was not included in the group of liver cirrhosis. Heterogeneous pattern of hepatic enhancement was observed on contrast CT scans^24^,^25^. Fortunately, clinical presentations and ultrasonography can easily differentiate cardiac cirrhosis from HSOS.

## Conclusion

In conclusion, the patients with PAs-induced HSOS display distinct radiologic features: patchy enhancement and heterogeneous hypoattenuation in portal phase of contrast-enhance CT; and contrast-enhanced CT possess better performance in the diagnosis of HSOS compared with various parameters of Seattle criteria, which revealed contrast-enhanced CT provided an effective method for diagnosing PAs-induced HSOS. Further studies should be performed to investigate the mechanism for CT imaging findings and the correlation between CT imaging and the prognosis.

## Methods

### Patients selection

Approval for this retrospective study was obtained from our college ethics committee, and the requirement for informed consent was waived. This retrospective study included 85 HSOS patients and the 222 controls. 85 consecutive HSOS patients who were admitted to our hospital from January 1, 2008, to Oct 31, 2015; 80 patients with HSOS were caused by gynura segetum, 5 patients were diagnosed as HSOS after HSCT (Fig. 1). For HSOS/VOD after HSCT, the patients satisfied the Seattle criteria. Two of three findings must occur within 20 days of transplantation: bilirubin >2 mg/dL (1 mg/dl = 17.1 umol/L), hepatomegaly or right upper quadrant pain, ascites or greater than 2% weight gain due to fluid accumulation^15^. For HSOS/VOD induced by gynura segetum, diagnostic criteria was described as previous study^4^,^6^,^8^,^26^.

Diagnostic criteria were (i) the patients met the modified Seattle criteria for HSOS characterized by hyperbilirubinemia, hepatomegaly and weight gain due to fluid accumulation; (ii) a Roussel Uclaf Causality Assessment Method (RUCAM) score 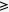 5; and (iii) a history of ingestion of gynura segetum. Inclusion criteria for patients recruitment were as follows: (A) patients with HSOS were caused by gynura segetum; (B) the patients had undergone contrast CT scan and the image data of CT were obtained. Exclusion criteria were: (A) the patients with HSOS who received HSCT; (B) HSOS patients caused by gynura segetum did not receive contrast CT; (C) the HSOS patients induced by gynura segetum had undergone contrast CT, but the image data were not obtained from a picture archiving and communication systems (PACS) (Fig. 1).

PAs-induced HSOS should be discriminated from other causes of hepatic venous outflow obstruction including Budd-Chiari syndrome (BCS) and congestive heart failure. However, most of the patients with congestive heart failure did not receive CT scan in our hospital. Additionally, the ascites resulted from portal hypertension is an important landmark of PAs-induced HSOS; thus, PAs-induced should be differentiated from cirrhotic patients with ascites or previous episodes of ascites. Therefore, the controls for gynura segetum-induced HSOS (Fig. 1) included Budd-Chiari syndrome and liver cirrhosis. Budd-Chiari syndrome is defined as hepatic venous outflow obstruction at any level from the small hepatic veins (HVs) to the junction of the inferior vena cava (IVC) and the right atrium, regardless of the cause of obstruction^1^,^27^,^28^. Outflow obstruction caused by HSOS and cardiac disorders is excluded from this definition. 57 consecutive patients with BCS were enrolled. The diagnosis of BCS was confirmed by clinical manifestations, by laboratory tests, by imaging modalities (ultrasonography, CT, MRI, or angiography). Digital subtraction angiography was performed in all BCS patients enrolled in our study. BCS is classified according to its location: HVs, IVC, and combined obstruction of HVs and IVC. 14 patient with BCS had obstruction in HVs, 26 patients had lesions of the IVC, 17 patients had mixed type with lesions of the IVC and the HVs^28^,^29^. Consecutive 165 patients with liver cirrhosis were enrolled in our study. The diagnosis of liver cirrhosis was based on a history of chronic liver disease, physical exam, and/or laboratory abnormalities and, importantly, the presence of signs of cirrhosis and/or portal hypertension (ascites, splenomegaly, and collateral circulation)^30^. Since all the patients with PAs-induced HSOS had ascites, the cirrhotic patients with ascites or previous episodes of ascites were enrolled in parallel with HSOS. Of the 165 patients, the cause of liver cirrhosis was hepatitis B in 122, hepatitis C in 23, alcoholic in 13, autoimmune hepatitis in 2 and primary billiary cirrhosis in 2, unknown cause in 3.

### Data collection

Data collected from medical records were tabulated in a database. The pertinent data included: (1) demographic data; (2) presenting symptoms and signs; (3) laboratory results of blood test; (4) imaging findings; (5) the results of ascitic examination (cell count and differential, total protein, albumin, cytology, lactate dehydrogenase, cholesterol, AFB smear, adenosine deaminase); (6) hospital courses; (7) pathological changes.

### Imaging Techniques and imaging analysis

All CT examinations were performed with one of the following three scanners: 64-detector spiral row scanner (Somatom Definition AS, Siemens, Germany; detector collimation, 64 × 0.6 mm; reconstruction, 5-mm section thickness; 100 kVp; and 126 mAs), 32-detector row dual-source CT scanner (Somatom Definition, Siemens, Germany; detector collimation, 32 × 0.6 mm; reconstruction, 5-mm section thickness; 120 kVp; and 244 mAs), 320-detector row dynamic volume CT (Aquilion ONE 640, Toshiba, Japan; detector collimation, 160 × 0.5 mm; reconstruction, 5-mm section thickness; 100 kVp; and 72 mAs). A total of 1.5 ml per kilogram of body weight of nonionic contrast agent (iohexol [GE Healthcare Co. Ltd, China], lopromide injection [Bayer Healthcare Co. Ltd, China], loversol injection [Hengrui Medicine Co. Ltd, China) was injected at a rate 3 mL/sec by an automatic power injector. Images were collected in arterial, portal venous, and equilibrium phases, respectively with 20–30 seconds, 45–60 seconds, and 120–180 seconds start delays after initiation of the contrast medium injection). An optimal timing for arterial phase imaging was determined using a bolus-tracking technique or a test-bolus injection technique. CT scan data were available on PACS and all images were reviewed at a PACS monitor. All CT examinations were randomized and retrospectively evaluated by two experienced abdominal radiologists, independently. the following abnormal findings were assessed, i.e., hepatomegaly (>18 cm; craniocaudally)^4^, gallbladder wall thickening (>3 mm)^4^, splenomegaly, ascites, pleural effusion, regenerative nodules, patchy enhancement (Fig. 2), heterogeneous hypoattenuation (Fig. 2), homogeneous in equilibrium phase, hepatic vein narrowing (the diameter of main right hepatic vein in hepatic venous phase images <1.5 mm)^4^, portosystemic collateral circulation. Furthermore, in order to assess the extent of heterogeneous hypoattenuation, we classified the severity of heterogeneous hypoattenuation on a 4-point ordinal scale in portal phase: grade A indicated homogenous parenchymal attenuation or homogeneous with negligible heterogeneity; score B, mild; score C, moderate; and score D, severe (Fig. 3).

### Statistical analysis

The interobserver agreement between the two radiologists for classifying the severity of heterogeneous hypoattenuation was determined using kappa (κ) statistics. The level of agreement was defined as follows: poor, κ-values of 0.20 or less; fair, κ-values of 0.21–0.40; moderate, κ-values of 0.41–0.60; good, κ-values of 0.61–0.80; and very good, κ-values of 0.81–1.00. Statistical analyses were performed using SPSS version 17.0 (SPSS Inc., Chicago, Illinois, USA). Continuous variables are presented as means and standard deviation and categorical variables as numbers and percentage. A *P* value less than 0.05 was considered indicative of a significant difference.

## Additional Information

**How to cite this article**: Kan, X. *et al.* Diagnostic performance of Contrast-enhanced CT in Pyrrolizidine Alkaloids-induced Hepatic Sinusoidal Obstructive Syndrome. *Sci. Rep.* **6**, 37998; doi: 10.1038/srep37998 (2016).

**Publisher's note:** Springer Nature remains neutral with regard to jurisdictional claims in published maps and institutional affiliations.

## Figures and Tables

**Table 1:** Baseline Characteristics of the Patients and Laboratory Tests.

	HSOS (n = 71)	BCS (n = 57)	Liver cirrhosis (n = 165)
Age, years (mean ± SD)	56.48 ± 11.84	44.63 ± 10.99**	51.40 ± 11.87**
Gender, *n* (M/F)	48/23	38/19	111/54
Clinical manifestation			
abdominal distension	100% (71/71)	68.42% (39/57)**	69.09% (114/165)**
right upper quadrant pain	61.97% (44/71)	17.54% (10/57)**	23.64% (39/165)**
weight gain	11.59% (8/69)	8.77% (5/57)	9.09% (15/165)
edema	36.62% (26/71)	38.60% (22/57)	36.36% (60/165)
Laboratory tests			
Erythrocytes, 10^12^/L (Mean ± SD)	4.54 ± 0.64	4.35 ± 0.82	3.12 ± 1.13**
HGB, g/L (Mean ± SD)	140.20 ± 20.54	127.55 ± 23.91**	106.28 ± 25.53**
leukocyte, 10^9^/L (Mean ± SD)	6.93 ± 2.76	4.83 ± 2.67**	4.61 ± 3.35**
PLT, ×10^9^/L (Mean ± SD)	117.67 ± 64.60	117.89 ± 76.58	111.31 ± 183.75
ALT, U/L (Mean ± SD)	132.24 ± 155.83	41.17 ± 70.11**	72.60 ± 91.39**
AST, U/L (Mean ± SD)	148.00 ± 161.18	44.79 ± 45.69**	88.89 ± 75.09**
ALP, U/L (mean ± SD)	177.91 ± 119.59	111.36 ± 49.75**	118.61 ± 56.44**
γ-GT, U/L (mean ± SD)	172.27 ± 128.21	96.79 ± 71.69**	83.42 ± 107.89**
Total bilirubin, μmol/L (mean ± SD)	69.81 ± 69.16	32.94 ± 25.59**	43.20 ± 55.78**
Albumin, g/L (mean ± SD)	30.76 ± 6.05	36.15 ± 6.52**	31.00 ± 6.56**
PT, S (Mean ± SD)	17.58 ± 2.87	15.64 ± 1.96**	17.55 ± 3.53
INR, (Mean ± SD)	1.49 ± 0.32	1.28 ± 0.21**	1.48 ± 0.41
Urea, mmol/L (Mean ± SD)	7.01 ± 3.76	5.16 ± 2.65**	5.79 ± 3.40*
Creatinine, μmol/L (Mean ± SD)	90.09 ± 44.66	61.84 ± 16.62**	68.85 ± 33.65**

Note: Normal ranges: erythrocytes: 3.0–5.5 × 10^12^/L; hemoglobin: 110–160 g/L; leukocyte: 4–10 × 10^9^/L; platelet: 100–300 × 10^9^/L; alanine aminotransferase (ALT): 5–35 U/L; aspartate aminotransferase (AST): 8–40 U/L; alkaline phosphatase (ALP) 40–150 U/L; albumin: 35–55 g/L; total bilirubin: 5.1–19 μmol/L; γ-glutamyl transpeptidase (γ-GT) 7–32 U/L; prothrombin time(PT): 11–16 S; Urea: 3.2–7.1 mmol/L; creatinine: 44–106 μmol/L. *Significant difference compared with HSOS (P < 0.05), **Significant difference compared with HSOS (P < 0.01).

**Table 2:** Summary of the CT features of HSOS caused by *gynura segetum* and the controls during initial presentation.

Variable	HSOS n/N(%)	BCS n/N(%)	Liver cirrhosis n/N(%)
hepatomegaly	56/71 (78.87)	27/57 (47.37)	10/165 (6.06)
gallbladder wall thickening	60/69 (86.96)*	21/57 (36.84)	96/161 (59.63)**
splenomegaly	18/71 (25.35)	45/57 (78.95)	135/161 (83.85)♯
asites	71/71 (100)	40/57 (70.18)	111/165 (67.27)
pleural effusion	50/71 (70.42)	11/57 (19.30)	50/165 (30.30)
regenerative nodules	4/71 (5.63)	8/57 (14.04)	28/165 (16.97)
patchy liver enhancement	66/71 (92.96)	16/57 (28.07)	0/165
heterogeneous hypoattenuation	71/71 (100)	11/57 (19.30)	0/165
A	0	46 (80.70)	165
B	12 (16.90)	4 (7.02)	0
C	26 (36.62)	6 (10.53)	0
D	33 (46.48)	1 (1.75)	0
homogeneous in equilibrium phase	6/71 (8.45)	8/16 (50%)	—
hepatic vein narrowing	62/71 (87.32)	25/57 (43.86)	4/165 (2.42)
portosystemic collateral circulation	19/71 (26.76)	49/57 (86.96)	112/165 (67.88)
esophageal and gastric varices	13/71 (18.31)	32/57 (56.14)	108/165 (65.45)
intrahepatic portosystemic shunts	4/71 (5.63)	44/57 (77.19)	18/165 (10.91)
spleno renal shunts	2/71 (2.82)	8/57 (14.04)	4/165 (2.42)
periumbilical veins	9/71 (12.68)	10/57 (17.54)	35/165 (21.21)

^*^Indicated that 2 of the patients with HSOS or liver cirrhosis had received cholecystectomy.

^**^Indicated 4 of cirrhotic patients had received cholecystectomy.

^#^Indicated 4 of cirrhotic patients had received splenectomy.

**Table 3:** Diagnostic performance of various parameters of Seattle criteria and radiologic signs of contrast-enhanced CT.

Variables	Sensitivity (%)	Specificity (%)	PPV (%)	NPV (%)	Accuracy (%)
Ascites	100	31.98 (25.84–38.12)	31.98 (0.2584–0.3812)	100	48.46 (42.74–0.5418)
Jaundice	50.7 (39.07–62.33)	61.93 (55.48–68.38)	30.25 (22.00–38.5)	79.41 (73.33–85.49)	59.17 (0.5350–0.6484)
Hepatomegaly	78.87 (69.37–88.37)	83.33 (78.43–88.23)	60.22 (50.27–70.17)	92.5 (88.85–96.15)	82.25 (77.87–86.63)
Right upper quadrant pain	61.97 (50.68–73.26)	77.93 (72.47–83.39)	47.31 (37.16–57.46)	86.5 (81.76–91.24)	74.06 (69.04–79.08)
Weight gain	11.59 (7.79–15.39)	90.99 (87.22–94.76)	28.57 (11.86–45.28)	76.81 (71.71–81.91)	72.16 (67.01–77.31)
Edema	36.62 (25.41–0.47.83)	62.9 (56.53–69.27)	24.07 (16.01–32.13)	75.54 (69.33–81.75)	56.51 (50.82–62.20)
Contrast-enhanced CT					
Patchy liver enhancement	92.96 (87.01–98.91)	92.79 (89.39–96.19)	80.49 (71.91–89.07)	97.63 (95.58–99.68)	92.83 (89.88–95.78)
Heterogeneous hypoattenuation	100	95.05 (92.20–9790)	86.59 (79.21–93.97)	100	96.25 (94.07–98.43)

Note: PPV = positive predictive value, NPV = negative predictive value data are presented as the median and 95% confidence interval in parentheses.
